# Prevalence and clinical correlates of suicidal ideation and aggression in patients with chronic schizophrenia: large-scale, cross-sectional study

**DOI:** 10.1192/bjo.2026.11999

**Published:** 2026-05-25

**Authors:** Ruomei Fan, Rongrong Zhu, Qihui Guo, Gang Rao, Dongmei Wang, Xiang-Yang Zhang

**Affiliations:** Affiliated Psychological Hospital of Anhui Medical University, Anhui Medical University, Hefei Fourth People’s Hospital, Hefei, China; State Key Laboratory of Cognitive Science and Mental Health, Institute of Psychology, Chinese Academy of Sciences, Beijing, China; Department of Psychology, University of Chinese Academy of Sciences, Beijing, China; Department of Psychological and Cognitive Sciences, Tsinghua University, Beijing, China

**Keywords:** Schizophrenia, aggression, suicidal ideation

## Abstract

**Background:**

Aggression has been closely linked to suicidal ideation in schizophrenia (SCZ). However, whether its correlates differ by suicidal ideation status remains unclear.

**Aims:**

This study aimed to assess the prevalence of aggression and compare the clinical correlates and predictive models of aggression in chronic SCZ patients with and without suicidal ideation.

**Method:**

A total of 659 chronic SCZ patients were recruited from 10 psychiatric hospitals in China. A brief interview assessed the presence of suicidal ideation in patients. Participants were assessed using the Insomnia Severity Index, Hamilton Depression Rating Scale, Modified Overt Aggression Scale and Positive and Negative Syndrome Scale.

**Results:**

SCZ patients with suicidal ideation exhibited a higher prevalence of aggression than those without (39.46 *v*. 22.15%, *p* < 0.001). Regardless of suicidal ideation status, patients with aggression were younger and presented with more severe clinical symptoms across multiple domains, except for negative symptoms which showed differential associations between groups. Among patients with suicidal ideation, those exhibiting aggression had a higher proportion of females and lower body mass index. Logistic regression analysis indicated that, in patients without suicidal ideation, male gender was an independent risk factor for aggression (odds ratio 1.856) whereas milder negative symptoms acted as a protective factor. In patients with suicidal ideation, positive and general psychopathology symptoms were positively associated with aggression whereas negative symptoms retained a protective effect.

**Conclusions:**

Our findings suggest that aggression is more prevalent in patients with suicidal ideation, and its clinical correlates differ significantly based on suicidal ideation status. Stratified assessment of aggression risk considering suicidal ideation is warranted.

Schizophrenia (SCZ) is a severe mental illness. Over the past three decades the global prevalence, incidence and disability-adjusted life years for SCZ have been steadily increasing. This places an immense burden on families, society and nations.^1^

In addition to core impairments in perception, thought, emotions, behaviour and cognition, aggression in individuals with SCZ warrants significant clinical attention.^2^ The estimated prevalence of aggression among SCZ patients is 33.3%, and it is associated with numerous adverse outcomes including serious harm to others, excessive treatment burdens, prolonged hospitalisations and higher readmission rates.^3^ Concurrently, suicidal ideation represents a significant clinical concern in chronic SCZ.^4^ Suicide is a leading cause of death in SCZ patients, with a mortality rate tenfold higher than that observed in the general population.^5^

A recent systematic review synthesising evidence from the past 15 years found that the vast majority of included studies (6 out of 8) supported a significant positive correlation between aggression and suicidal ideation in SCZ patients.^6^ This association may stem from shared neurobiological underpinnings, such as dysfunction within the serotonin system.^7,8^ Therefore, elucidating the prevalence, associated factors and interrelationship of aggression and suicidal ideation is crucial for effective management of aggression risk in patients with SCZ, particularly in developing countries.^3^

Depressive symptoms are common among individuals with SCZ, with a reported comorbidity rate of 28.6%.^9^ Extensive research has established depressive symptoms as a key risk factor for suicidal ideation. Previous studies have also demonstrated a significant association between depressive symptoms and aggression in both adolescent and adult populations.^10–12^ Sleep disturbance is another critical issue in SCZ patients:^13,14^ reduced sleep duration and poor sleep quality are associated with increased aggression and suicidal ideation.^14–16^ Furthermore, the core psychopathological symptoms of SCZ are fundamental to understanding patient behaviour and clinical outcomes.^6,17^

However, it remains unclear whether the clinical and demographic factors associated with aggression differ between SCZ patients with and without suicidal ideation. The primary aim of this study was therefore twofold: first, to assess the prevalence of aggression in chronic SCZ patients stratified by suicidal ideation status; and second, and most importantly, to identify and compare the independent clinical correlates and predictive models for aggression between patients with and without suicidal ideation. To the best of our knowledge, this is the first study to specifically investigate and compare the predictive models of aggression in chronic SCZ patients stratified by the presence of suicidal ideation.

## Method

### Study sample and design

A total of 659 chronic SCZ patients were recruited from 10 psychiatric hospitals across China. The study protocol was approved by the Institutional Review Board of the Institute of Psychology, Chinese Academy of Sciences (approval no. H18031). All participants provided written informed consent.

The inclusion criteria were as follows: (a) aged between 16 and 65 years; (b) a diagnosis of SCZ confirmed by two experienced psychiatrists using the Structured Clinical Interview for DSM-IV; (c) a documented illness duration of at least 1 year; and (d) a stable dose of antipsychotic medication for at least 6 months prior to study enrolment. The exclusion criteria were: (a) a history of substance or alcohol abuse, except for nicotine use; (b) current pregnancy or lactation; and (c) a diagnosis of any organic brain diseases or severe physical illnesses that could interfere with assessment.

### Demographic characteristics

A standardised questionnaire was designed and administered to collect demographic information on age, gender, years of education and body mass index (BMI). Clinical information was also obtained, encompassing family psychiatric history, onset age and current antipsychotic medication regimen. Gender was recorded based on biological gender at birth (male/female). The type and daily dosage of each antipsychotic medication were recorded in detail, and the total daily chlorpromazine equivalent dose was calculated.

### Assessment of suicidal ideation and aggression

A brief, investigator-designed clinical interview was conducted to ascertain the presence of suicidal ideation. Each participant was asked: ‘Have you ever had thoughts of killing yourself (suicidal ideation) or have you ever engaged in any suicidal behaviour?’ Based on their responses, patients were categorised into one of three groups: those reporting current or past suicidal ideation; those reporting suicidal ideation but no history of suicidal behaviour; and those with a history of suicidal behaviour. For the primary analysis of this study, patients were ultimately dichotomised into two groups: those with any lifetime history of suicidal ideation (including those with suicidal behaviour) and those without any history of suicidal ideation. Patients reporting a history of suicidal behaviour were considered to have had suicidal ideation.

Aggression was assessed using the Modified Aggression Scale (MOAS).^18^ MOAS is a semi-structured interview that evaluates four clusters of aggression: verbal aggression, aggression against property, auto-aggression and physical aggression against others. Each subscale is rated from 0 (no aggression) to 4 (most severe aggression), then a weighted total score is calculated. For the purpose of this study, participants were classified into two groups: a non-aggression group (MOAS total score 0) and an aggression group (MOAS total score >0, indicating that at least one rated item was greater than 0). The aggression group was defined as participants with a MOAS score >0, encompassing any observable aggressive incident. This broad cut-off was selected to capture the full spectrum of aggression, including minor verbal or physical threats that may represent early signs of agitation, consistent with prior research focusing on prodromal aggression.

### Clinical measurements

The Insomnia Severity Index (ISI) was used to assess the severity of insomnia symptoms over the preceding 2 weeks.^19^ This 7-item scale is scored from 0 to 28, with a score of 8 or higher indicating clinically significant insomnia. Higher total scores reflect greater perceived insomnia severity.

The 17-item Hamilton Depression Rating Scale (HAMD) was administered to assess the severity of depressive symptoms.^20^ Higher total scores on this scale indicate more severe depressive symptomatology.

The Positive and Negative Syndrome Scale (PANSS) was used to assess psychopathological symptoms in SCZ patients.^21^ This 30-item scale yields a total score and 3 subscale scores: Positive Symptoms (PANSP, 7 items), Negative Symptoms (PANSN, 7 items) and General Psychopathology (PANSG, 16 items). Higher scores on PANSS and its subscales indicate greater severity of psychiatric symptoms.

### Statistical analysis

Descriptive statistics were calculated for all study variables. Group comparisons (suicidal ideation versus non-suicidal ideation) for categorical variables were conducted using chi-square tests; independent-samples *t*-tests were used to compare continuous variables between these groups.

To identify factors independently associated with the presence of aggression (MOAS > 0), separate binary logistic regression analyses were performed for the subgroups of patients with and without suicidal ideation. The backward stepwise elimination method was employed. Variables entered into the initial models included demographic factors (e.g. gender, age) and key clinical scale scores (ISI total, HAMD total, PANSP, PANSN, PANSG), with aggression status (yes/no) as the dependent variable.

The discriminative ability of the significant variables identified in the final regression models, both individually and in combination, was evaluated by calculating the area under the receiver operating characteristic curve (AUC).

All statistical analyses were performed using SPSS version 26.0 (IBM Corp., Armonk, New York, USA; https://www.ibm.com/products/spss-statistics), running on Windows 11.

## Results

### Prevalence of aggression in SCZ patients with and without suicidal ideation

This study included a total of 659 patients with chronic SCZ. Among these individuals, 185 (28.07%) reported a history of suicidal ideation (suicidal ideation+ group) and 474 (71.93%) did not (suicidal ideation− group). The prevalence of aggression was significantly higher in the suicidal ideation+ group (39.46%, *n* = 73) compared with the suicidal ideation− group (22.15%, *n* = 105), and this difference was statistically significant (*χ* ^2^ = 20.218, *p* < 0.001).

As detailed in Table 1, compared with suicidal ideation− patients, those in the suicidal ideation+ group were significantly younger, had an earlier age of illness onset and included a higher proportion of females. Clinically, the suicidal ideation+ group exhibited more severe symptom profiles, as evidenced by significantly higher scores on ISI, HAMD, PANSS total score, PANSP and PANSG (all *p* < 0.05). Consistent with the prevalence data, total scores on MOAS were also significantly higher in the suicidal ideation+ group compared with the suicidal ideation− group (*p* < 0.001).

**Table 1:** Characteristics of patients with chronic schizophrenia, grouped by suicidal ideation

Variables	Without suicidal ideation (*n* = 474)	With suicidal ideation (*n* = 185)	*t* 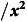	*P*-value
Gender, *n* (%)			5.180^a^	**0.023**
** **Male	338 (71.31)	115 (62.16)		
** **Female	136 (28.69)	70 (37.84)		
Age, years	42.47 ± 12.641	38.72 ± 11.541	3.647^b^	**<0.001**
Years of education	9.40 ± 2.871	9.66 ± 2.922	−1.014^b^	0.311
Family history, *n* (%)			3.058^a^	0.080
** **Yes	82 (17.30)	43 (23.24)		
** **No	392 (82.70)	142 (76.76)		
BMI	23.96 ± 3.973	24.00 ± 4.239	−0.105^b^	0.917
Onset age, years	24.27 ± 6.550	23.10 ± 6.491	2.061^a^	**0.040**
Antipsychotic dosage	417.00 ± 229.017	452.48 ± 242.198	−1.716^b^	0.087
ISI	3.23 ± 4.351	4.78 ± 5.349	−3.519^b^	**0.001**
HAMD	5.22 ± 4.196	7.728 ± 4.655	−6.378^b^	**<0.001**
PANSS	77.47 ± 21.609	84.84 ± 23.33	−3.718^b^	**<0.001**
** **PANSP	17.11 ± 7.184	19.47 ± 7.001	−3.859^b^	**<0.001**
** **PANSN	23.18 ± 7.492	23.52 ± 7.624	−0.516^b^	0.606
** **PANSG	37.22 ± 11.059	41.91 ± 12.059	−4.597^b^	**<0.001**
MOAS	1.06 ± 2.998	2.75 ± 5.289	−4.092^b^	**<0.001**
** **MOAS1	0.29 ± 0.677	0.53 ± 0.860	−3.378^b^	**<0.001**
** **MOAS2	0.27 ± 1.058	0.45 ± 1.433	−1.565^b^	**0.001**
** **MOAS3	0.07 ± 0.516	0.89 ± 2.623	−4.251^b^	**<0.001**
** **MOAS4	0.45 ± 1.994	0.89 ± 2.765	−1.976^b^	**<0.001**

BMI, body mass index; ISI, Insomnia Severity Index; HAMD, Hamilton Depression Rating Scale; PANSS, Positive and Negative Syndrome Scale; PANSP, PANSS Positive Symptoms; PANSN, PANSS Negative Symptoms; PANSG, PANSS General Psychopathology; MOAS, Manifest Aggression Scale; MOAS1, verbal aggression; MOAS2, aggression against property; MOAS3, aggression against oneself; MOAS4, physical aggression against others.

a. Chi-square test.

b. Independent samples *t*-test. Categorical variables (gender, family history) were analysed using chi-square test, while continuous variables (age, years of education, clinical scale scores, etc.) were analysed using independent samples *t*-test.

Bold font indicates statistical significance.

### Clinical characteristics of SCZ patients with and without aggression, stratified by suicidal ideation status

Table 2 presents the clinical characteristics of patients stratified by both suicidal ideation status and the presence of aggression.

**Table 2:** Characteristics of chronic schizophrenia patients with and without aggression, grouped by suicidal ideation

Variables	Without suicidal ideation				With suicidal ideation			
	Non-aggression	Aggression	*t* 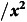	*p*-value	Non-aggression	Aggression	*t* 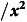	*p*-value
	*n* = 369	*n* = 105			*n* = 112	*n* = 73		
Gender, *n* (%)			0.076	0.783			6.753	**0.009**
** **Male	262 (71.00)	76 (72.38)			78 (69.64)	37 (50.68)		
** **Female	107 (29.00)	29 (27.62)			34 (30.36)	36 (49.32)		
Age, years	43.48 ± 12.768	38.90 ± 11.553	3.497	**0.001**	40.07 ± 12.419	36.64 ± 9.769	2.092	**0.038**
Years of education	9.34 ± 2.716	9.63 ± 3.366	−0.814	0.417	9.76 ± 2.816	9.50 ± 3.091	0.565	0.573
Family history, *n* (%)			0.116	0.733			1.117	0.291
** **Yes	65 (17.62)	17 (16.19)			29 (25.89)	14 (19.18)		
** **No	304 (82.38)	88 (83.81)			83 (74.11)	59 (80.82)		
BMI	23.89 ± 4.033	24.19 ± 3.763	−0.703	0.483	24.49 ± 4.421	23.25 ± 3.852	2.016	**0.045**
Onset age, years	24.44 ± 6.613	23.66 ± 6.318	1.111	0.268	23.48 ± 6.721	22.54 ± 6.124	0.981	0.328
Antipsychotic dosage	414.25 ± 233.318	426.68 ± 213.995	−0.515	0.607	452.98 ± 231.683	451.73 ± 259.144	0.033	0.973
ISI	2.65 ± 3.943	5.26 ± 5.073	−4.862	**<0.001**	3.59 ± 4.288	6.61 ± 6.258	−3.615	**<0.001**
HAMD	4.95 ± 4.198	6.18 ± 4.063	−2.717	**0.007**	6.77 ± 4.703	9.19 ± 4.206	−3.650	**<0.001**
PANSS	74.22	88.91	−6.238	**<0.001**	76.42	97.76	−6.952	**<0.001**
** **PANSP	20.532	21.504	−7.592	**<0.001**	21.799	19.458	−7.042	**<0.001**
** **PANSN	15.79 ± 6.630	21.72 ± 7.179	−0.785	0.433	16.86 ± 6.176	23.47 ± 6.290	−2.857	**0.005**
** **PANSG	23.04 ± 7.625	23.66 ± 7.017	−6.706	**<0.001**	22.28 ± 7.808	25.42 ± 6.964	−7.558	**<0.001**
	35.44 ± 10.444	43.47 ± 10.934			37.28 ± 11.188	49.01 ± 9.706		
MOAS	0 ± 0	4.80 ± 4.771	−10.31	**<0.001**	0 ± 0	6.97 ± 6.97	−9.232	**<0.001**

BMI, body mass index; ISI, Insomnia Severity Index; HAMD, Hamilton Depression Rating Scale; PANSS, Positive and Negative Syndrome Scale; PANSP, PANSS Positive Symptoms; PANSN, PANSS Negative Symptoms; PANSG, PANSS General Psychopathology; MOAS, Manifest Aggression Scale.

Bold font indicates statistical significance.

Among suicidal ideation− patients, individuals with aggression (Agg+, *n* = 105) were significantly younger and had higher HAMD total scores (*p* < 0.05) compared with those without aggression (Agg−, *n* = 369). Additionally, Agg+ individuals showed significantly higher scores on ISI, PANSS total score, PANSP and PANSG (all *p* < 0.001).

Among suicidal ideation+ patients, Agg+ (*n* = 73) contained a significantly higher proportion of females compared with Agg− (*n* = 112, *p* < 0.05). Furthermore, Agg+ had lower age, lower body mass index (BMI) and higher scores on PANSN (all *p* < 0.05) compared with Agg−. Agg+ also demonstrated significantly higher ISI and HAMD and PANSS total scores and PANSP and PANSG scores (all *p* < 0.001).

When comparing the two aggression subgroups directly, patients in the suicidal ideation+/Agg+ group (*n* = 73) exhibited more severe psychopathology than those in the suicidal ideation−/Agg+ group (*n* = 105), particularly in PANSS total score (97.76 *v*. 88.91), PANSN score (25.42 *v*. 23.66) and HAMD score (9.19 *v*. 6.18), suggesting that the co-occurrence of suicidal ideation and aggression is associated with a more severe clinical profile.

A sensitivity analysis using MOAS ≥ 4 confirmed the robustness of core findings: Agg+ remained younger, with higher scores on ISI, HAMD, PANSS total, PANSP and PANSG (all *p* < 0.05). Subgroup stratification by suicidal ideation showed no material changes to key conclusions.

### Clinical correlates of aggression in SCZ patients stratified by suicidal ideation status

To identify independent clinical correlates of aggression, separate binary logistic regression analyses were performed for suicidal ideation− and suicidal ideation+ patients.

**Fig. 1:**
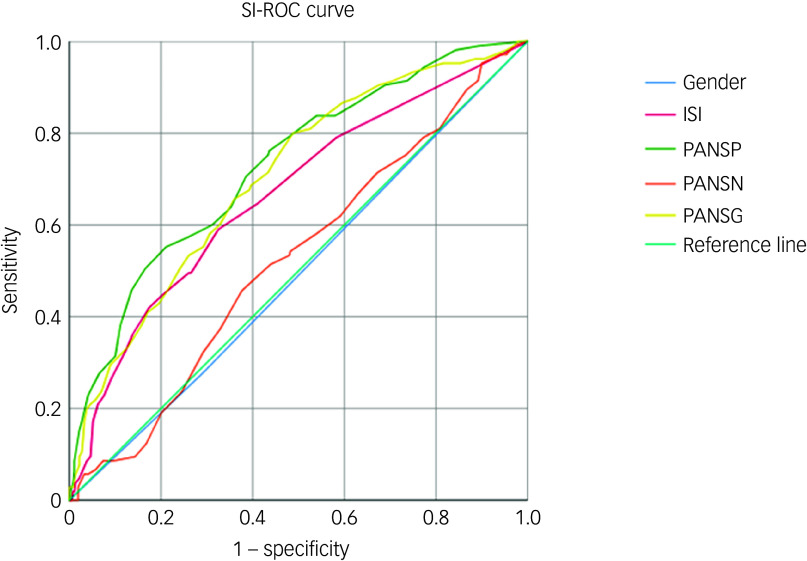
Receiver operating characteristic (ROC) curve in schizophrenia patients without suicidal ideation (SI). Area under the curve for those without suicidal ideation-ROC curves (regression model 0.783; gender 0.493; ISI 0.665; PANSP 0.728; PANSN 0.524; PANSG 0.703). ISI, Insomnia Severity Index; PANSP, Positive and Negative Syndrome Scale Positive Symptoms; PANSN, Positive and Negative Syndrome Scale Negative Symptoms; PANSG, Positive and Negative Syndrome Scale General Psychopathology.

In suicidal ideation− patients, the regression model included gender, ISI total score and all three PANSS subscale scores (PANSP, PANSN, PANSG) as independent variables, with the presence of aggression as the dependent variable. The results showed that gender (*B* = 0.618, *p* = 0.032, odds ratio 1.856, 95% CI 1.055–3.265), ISI (*B* = 0.073, *p* = 0.004, odds ratio 1.076, 95% CI 1.023–1.132), PANSP (*B* = 0.079, *p* < 0.001, odds ratio 1.083, 95% CI 1.035–1.132) and PANSG (*B* = 0.066, *p* < 0.001, odds ratio 1.068, 95% CI 1.031–1.107) were positively and independently correlated with the presence of aggression. Conversely, PANSN (*B* = −0.069, *p* = 0.03, odds ratio 0.933, 95% CI 0.892–0.977) was negatively correlated with aggression, indicating a protective effect (Table 3). Receiver operating characteristic curve analysis was used to assess the predictive performance. AUC values for individual significant predictors were as follows: gender 0.493, ISI 0.665, PANSP 0.728, PANSN 0.524, PANSG 0.703 (Fig. 1). The combined regression model incorporating these variables yielded an AUC of 0.783.

**Table 3:** Binary logistic regression model to predict the presence of aggression

Variables	Without suicidal ideation				With suicidal ideation			
	*B*	Wald x2	*p*	Odds ratio (95% CI)	*B*	Wald x2	*p*	Odds ratio (95% CI)
Gender	0.618	4.605	**0.032**	1.856 (1.055, 3.265)				
ISI	0.073	8.125	**0.004**	1.076 (1.023, 1.132)				
PANSS								
** **PANSP	0.079	12.216	**<0.001**	1.083 (1.035, 1.132)	0.089	5.559	**0.018**	1.093 (1.015, 1.176)
** **PANSN	−0.069	8.732	**0.003**	0.933 (0.892, 0.977)	−0.079	5.112	**0.024**	0.924 (0.862, 0.989)
** **PANSG	0.066	12.921	**<0.001**	1.068 (1.031, 1.107)	0.107	14.622	**<0.001**	1.113 (1.054, 1.176)

ISI, Insomnia Severity Index; PANSS, Positive and Negative Syndrome Scale; PANSP, PANSS Positive Symptoms; PANSN, PANSS Negative Symptoms; PANSG, PANSS General Psychopathology.

Bold font indicates statistical significance.

**Fig. 2:**
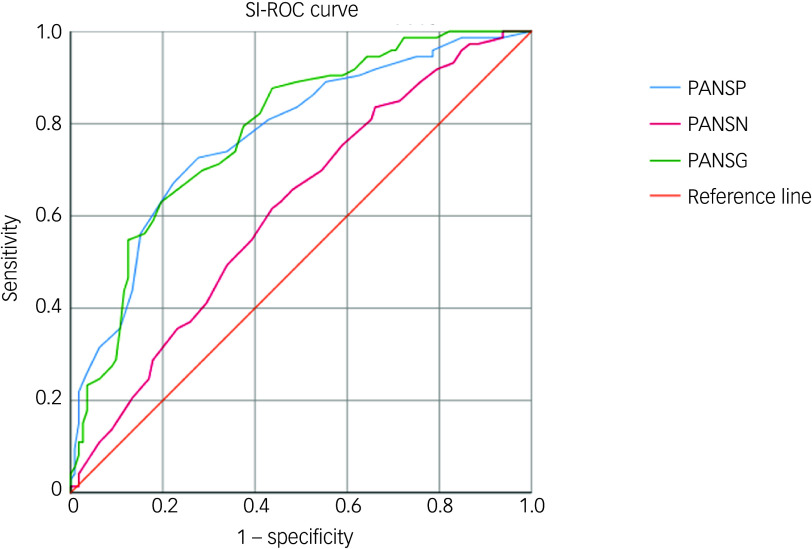
Receiver operating characteristic (ROC) curve in schizophrenia patients with suicidal ideation (SI). Area under the curve for those with suicidal ideation-ROC curves (regression model 0.825; PANSP 0.774; PANSN 0.615; PANSG 0.784). PANSP, Positive and Negative Syndrome Scale Positive Symptoms; PANSN, Positive and Negative Syndrome Scale Negative Symptoms; PANSG, Positive and Negative Syndrome Scale General Psychopathology.

In suicidal ideation+ patients, a binary logistic regression was performed with several variables, including all subscale scores of PANSS as independent variables and the presence of aggression as a dependent variable. The results showed that PANSP (*B* = 0.089, *p* = 0.018, odds ratio 1.093, 95% CI 1.015–1.176) and PANSG (*B* = 0.107, *p* < 0.001, odds ratio 1.113, 95% CI 1.054–1.176) were positively and independently correlated with the presence of aggression. Conversely, PANSN (*B* = −0.079, *p* = 0.024, odds ratio 0.924, 95% CI 0.862–0.989) was negatively correlated with aggression (Table 3). AUC values for the individual PANSS subscales were as follows: PANSP 0.774, PANSG 0.615, PANSN 0.784 (Fig. 2). The combined regression model for this subgroup achieved an AUC of 0.825.

## Discussion

### Prevalence of aggression in patients with and without suicidal ideation

Our study revealed a significantly higher prevalence of aggression among SCZ patients with suicidal ideation compared with those without (39.46 *v*. 22.15%). This finding aligns with a recent systematic review by Bravve et al, which concluded that most studies support a significant association between suicidal ideation and aggression in SCZ.^6^ Further corroboration comes from a large-scale study by Lin et al, which reported a bidirectional association, noting a higher detection rate of overt aggression in patients with severe mental disorders and suicidal ideation (39.3 *v*. 27.3%).^22^ Following multivariable adjustment, aggression was found to double the risk of suicide (odds ratio 2.008), underscoring suicidal ideation as a potent predictor of aggression. This further confirms that suicidal ideation is a strong predictor of aggression. Research on impulsivity provides additional support: Iancu et al found that, among male SCZ patients, those with high impulsivity traits had higher rates of current suicidal ideation and significantly higher aggression levels.^23^ Importantly, in their predictive model, aggression was an independent predictor of suicide risk (*β* = 0.36, *p* < 0.001), reinforcing the intrinsic link between these behaviours.

However, in a German retrospective study, Neuner et al found no significant difference in aggressive behaviour between SCZ patients with and without a history of suicide attempt/completion.^24^ This discrepancy may stem from methodological factors, including the use of routine clinical data leading to a less sensitive dichotomous measure of aggression, and limited statistical power due to a small end-point sample (*n* = 47). Therefore, this negative finding does not negate a potential conceptual relationship.

Neuroscientific evidence further substantiates a connection. Shared neurobiological disturbances, particularly dysfunctions in the serotonergic system (e.g. involving 5-HT1A and 5-HT2A receptors), have been implicated in both aggression and suicidality.^7,8^ Moreover, neuroimaging studies link aggression in SCZ to specific patterns of cerebral cortical thinning and altered brain activation,^25,26^ which may also represent neural vulnerabilities for suicidality.

Notably, existing literature has primarily focused on aggression in relation to broader suicidality or suicidal behaviour. The specific neurobiological and clinical mechanisms linking aggression specifically to suicidal ideation remain less clearly delineated and warrant further focused investigation.

### Common correlates of aggression

Our findings indicate that SCZ patients exhibiting aggression present with more severe clinical profiles, regardless of suicidal ideation status.

The association between sleep disturbance and aggression is well established.^15,27^ Recent studies in SCZ confirm that insomnia is closely linked to aggression, mediated through complex pathways involving factors including quality of life and depression.^13,28^ Neurobiologically, sleep deprivation impairs the function of the anterior cingulate gyrus, a prefrontal region involved in impulse and emotion regulation.^15^ Given the pre-existing frontal lobe deficits in SCZ,^29^ the interaction of these factors may heighten aggression risk.

Depressive symptoms, common across all stages of SCZ, are also linked to aggression.^2^ Studies in adolescents show that depressive symptoms can predict violence,^10^ and research in rural Chinese SCZ patients found that aggression correlated with depressive mood.^30^ A mediation model suggesting a chain (depression → insomnia → aggression → suicidal ideation) highlights the complex interplay of these risk factors,^28^ although precise causal mechanisms require clarification.

Consistent with the literature, our study found that aggressive patients, irrespective of suicidal ideation, had more severe overall psychopathology, particularly in positive and general psychopathology dimensions. Aggression is a marker for complex suicide/self-harm subgroups and correlates with higher PANSS total and positive scores,^31^ whereas general psychopathology is a core predictor of suicide risk.^23^

In summary, regardless of suicidal ideation status, aggressive SCZ patients exhibit more severe insomnia, depressive symptoms and overall psychotic symptoms – especially positive and general psychopathology. Monitoring these indicators is crucial for identifying individuals at risk for aggression.

### Differential correlates by suicidal ideation status

Our bivariate analyses suggested associations between aggression and factors including age (in non-suicidal ideation patients) and gender/BMI/affective symptoms (in suicidal ideation patients). However, logistic regression revealed fundamental differences in the independent clinical correlates of aggression based on suicidal ideation status, involving not only distinct risk factor combinations but also divergent roles of the same factors.

In patients without suicidal ideation, male gender was an independent risk factor for aggression (odds ratio 1.856). This aligns with research showing gender differences in symptom presentation, with aggression being more prevalent among men with psychosis.^32^ Biological mechanisms may contribute: for instance, studies link higher cholesterol and low-density lipoprotein levels to impulsivity in men with SCZ,^33^ and decades of animal research associate dopamine with aggression in males.^34^ Dopaminergic dysfunction in SCZ is directly linked to positive symptoms and aggression, and its impact on aggression may be more pronounced in male patients. The specific mechanisms involved require further study. Notably, the effect of gender was not significant in univariate analysis, possibly due to confounding by other clinical variables.

A key finding was the significant protective effect of negative symptoms against aggression in patients without suicidal ideation. Negative symptoms (e.g. blunted affect, avolition, anhedonia)^35^ are considered a core feature of SCZ. This state of reduced emotional responsiveness and motivation may decrease engagement with external triggers, thereby lowering the risk of overt aggression.^36^

In the model for patients with suicidal ideation, gender and insomnia were not independent predictors whereas general psychopathology remained significant. This suggests that subjective distress and agitation are primary drivers of aggression in this subgroup. Although the protective effect of negative symptoms persisted, and given the association between suicidal ideation and aggression, negative symptoms may inhibit outward-directed aggression without mitigating inwardly directed aggression (i.e. self-harm).

### Limitations

This study has several limitations. First, the cross-sectional design precludes causal inferences regarding the relationships among suicidal ideation, clinical correlates and aggression. Longitudinal studies are urgently needed to clarify the temporal and potentially causal pathways linking these factors in patients with chronic SCZ.

Second, the assessment of suicidal ideation relied on a single, non-standardised interview question rather than a validated instrument (e.g. Calgary Depression Scale item 9 or Beck Scale for Suicidal Ideation). This approach lacks established reliability and validity and may not capture the full spectrum or severity of suicidal ideation. Additionally, our assessment relied on lifetime history of suicidal ideation and did not capture current (e.g. past-month) ideation, which may have different clinical correlates from remote ideation. Future studies should employ standardised scales to provide a more robust assessment.

Third, as a clinician-rated tool, MOAS may be subject to reporting bias in in-patient settings. Staff might underreport minor incidents or, conversely, overreport behaviours due to increased vigilance. Future studies could incorporate objective behavioural monitoring or multi-rater assessments to address this limitation.

Fourth, several potential confounders (e.g. childhood trauma, psychosocial stressors, medication adherence) were not measured and may have influenced our findings.

Finally, because our sample consisted of chronic SCZ patients, the findings may not be generalisable to first-episode or treatment-resistant populations.

In conclusion, this study provides evidence that aggression is more prevalent in chronic SCZ patients with suicidal ideation, and that its clinical correlates differ significantly based on suicidal ideation status. Therefore, a stratified approach to aggression risk assessment, distinguishing between patients with and without suicidal ideation, is clinically indicated. Future research should aim to elucidate the mechanisms underlying these subgroup differences, which will be crucial for developing more targeted and effective interventions for aggression in SCZ.

## Data Availability

The data that support the findings of this study are publicly available. The corresponding author can be contacted upon reasonable request.
